# Susac’s syndrome as HIV-associated immune reconstitution inflammatory syndrome

**DOI:** 10.1186/1742-6405-10-22

**Published:** 2013-09-03

**Authors:** Francesca Ferretti, Simonetta Gerevini, Bruno Colombo, Manuela Testa, Monica Guffanti, Diego Franciotta, Gaetano Bernardi, Adriano Lazzarin, Paola Cinque

**Affiliations:** 1 Department of Infectious Diseases, San Raffaele Scientific Institute, Milan, Italy; 2 Unit of Neuroradiology, San Raffaele Scientific Institute, Milan, Italy; 3 Department of Neurology, San Raffaele Scientific Institute, Milan, Italy; 4 Department of Neurology, IRCCS, C. Mondino National Institute of Neurology, Pavia, Italy; 5 Department of Neurology, IRCCS Neurologic Institute Carlo Besta Institute, Milan, Italy

## Abstract

Susac’s Syndrome (SS) is an autoimmune endotheliopathy of cerebral, retinal and cochlear arterioles. We report of an HIV-infected woman who developed a first SS episode following a spontaneous reduction of plasma viral load and several relapses six years later, following initiation of combined antiretroviral therapy (cART). Corticosteroids and intravenous immunoglobulins alone did not control the disease, which improved after combined treatment with acyclovir and ganciclovir. SS onset in HIV infection and relapses during cART-induced immune reconstitution are consistent with the dysimmune nature of the disease. The response to anti-herpes drugs suggests a viral contribute in this case of SS.

## Introduction

Susac’s syndrome (SS) is a rare autoimmune microangiopathic disorder, affecting precapillary arterioles of the brain, retina and cochlea [1]. Until now, approximately 200 cases have been reported [2], mainly in women. Headache, focal neurological signs, deafness and ocular symptoms are the most common clinical manifestations, but do not necessary occur simultaneously. SS is almost never fatal [3], however it is characterized by spontaneous remissions and relapses that can only partially be controlled or delayed by immunosuppressive drugs and often lead to irreversible neurological sequelae and poor quality of life.

We here describe a case of SS in an HIV-infected woman, who developed a first episode following a spontaneous decrease of plasma viral load, and several relapses 6 years later, following introduction of combined antiretroviral therapy (cART), as likely expression of an immune reconstitution inflammatory syndrome (IRIS). Notably, the neurological picture was not controlled by corticosteroids and intravenous immunoglobulins alone, but only when acyclovir and ganciclovir were administered concomitantly, suggesting a possible role of herpes viruses in SS pathogenesis in this case.

## Case report

In September 2002, a 42 year-old woman with a 15-year history of untreated HIV-1 and hepatitis C virus infection was admitted to our Infectious Diseases Department with headache, facial paresthesias, amaurosis, hemianopsia, tinnitus and vertigo (Table 1). Blood CD4+ cells were 355/μL and plasma HIV-RNA level had unexplainably dropped from 270,000 to 2000 copies/mL in the previous 9 months, in absence of ART. Brain magnetic resonance imaging (MRI) showed T2-hyperintense lesions in the basal ganglia, bilateral subcortical and deep cerebral white matter and medium-posterior corpus callosum, some of which were gadolinium enhancing (images not available). Cerebrospinal fluid (CSF) analysis showed only mild pleocytosis and protein increase, fundus oculi examination a retinal vascular occlusion in the superior temporal regions, audiometric examination a neuro-sensorial left hypoacusia. The diagnosis was of cerebral vasculitis and suspected cytomegalovirus (CMV) retinopathy. Intravenous (i.v.) methylprednisolone and gancyclovir were administered (Table 1), followed by clinical resolution.

**Table 1:** Clinical, laboratory, neuroradiological findings and therapies for each Susac Syndrome episode

**Date**	**New neurological symptoms**	**Laboratory examinations**	**Brain MRI**	**Other examinations**	**Ongoing therapy (duration)**	**New therapy (duration)**	**Clinical Outcome**
**First episode** September 2002	Headache, facial paresthesias, hemianopsia, amaurosis, tinnitus, vertigo	Blood	T2-hyperintense Gd-enhancing lesions (brain)	FO and RFA: retinal branch occlusion. Auditory examination: initial left neurosensorial hypoacusia. VEP, AEP: normal	None	IV MEP 20 mg bid (3 days). IV GCV 5 mg/Kg bid (14 days)	Resolution
		CD4+:355/μL					
		VL: 2000 c/mL					
		VDRL and TPHA neg					
		CSF					
		Cells: 5/mL					
		Proteins: 89 gr/dL					
		Microbiology * neg					
		Viral genomes ** neg					
		VL<50 c/mL					
**First relapse** March 2008	Headache, facial, lingual, oral and hand paresthesias	Blood	Increased T2 hyperintensity of old lesions; new T2 hyperintense non Gd-enhancing lesion (brain) (Figure 1a)	EEG: focal slow abnormal activity in the left temporal region	cART: TDF, FTC, ATV (6 weeks)	Oral PDN 50 mg qd (5 days), then 25 mg qd (3 days). Stop cART	Worsening
		CD4+: 260/μL					
		VL<50 c/mL					
		VDRL and TPHA neg					
**First relapse, follow-up (SS diagnosis)** April 2008	Left hemiparesis, acute left hypoacusia	CSF	Further increased T2 hyperintensity of old lesions; new T2 hyperintense non Gd-enhancing lesions (cerebellum) (Figure 1b)	Visual field: central scotoma of right eye, arcuate scotoma in the superior and inferior field of left eye. FO: right retinal vasculopathy. RFA: acute bilateral retinal vasculitis with reduced perfusion. VEP: absent response of right eye, reduced response of left eye; AEP: mixed bilateral hypoacusia.	None	IV MEP 1 g qd (5 days), then oral PDN 50 mg qd (10 days). cART: TDF, FTC, ATV	Transient improvement
		Cells: 1/mL					
		Proteins: 23 g/dL					
		Viral genomes*: neg					
		Oligoclonal bands: neg					
		IgG: 64 mg/dL					
		Albumin ratio: 4.52					
		Intrathecal HSV-1/2, VZV and CMV-specific IgG synthesis: neg					
**Second relapse** April 2008	Blurred vision, hallucinations, gait and balance deficit	Blood c-ANCA, p-ANCA, anti cardiolipin, anti-beta 2-gp, LA and ANA: neg	New T2 hyperintense lesions with mild Gd-enhancement		cART: TDF, FTC, ATV (3 weeks)	IV MEP 1 g qd (6 days), then oral PDN 75 mg qd	Transient improvement
**Third relapse** May 2008	Worsening of previous symptoms	Blood	New Gd-enhancing lesions (brain and brain stem) (Figure 1c)		cART: TDF, FTC, ATV (6 weeks). Oral PDN 75 mg qd (5 days)	IV Ig 15.5 g qd (5 days). IV MEP 40 mg bid (6 days)	No changes
		CD4+: 113/μL					
		VL<50 c/mL					
**Third relapse, follow-up** May 2008	Persistence of symptoms	CSF			cART: TDF, FTC, ATV (8 weeks). IV MEP 40 mg bid (6 days)	IV MEP 1 g qd (3 days), then oral PDN 150 mg qd, tapered to 5 mg in 16 weeks. IV GCV 5 mg/Kg bid (14 days), then oral V-GCV 450 mg qd (4 weeks). IV ACV 15 mg/Kg tid (14 days), then oral ACV 800 mg q5h (3 weeks)	Improvement
		Cells: N.A.					
		Proteins: 172 g/dL					
**Fourth relapse** November 2008	Vertigo, visus deficit	Blood	New small Gd-enhancing lesions (cerebellum) **(**Figure 1d)		cART: TDF, 3TC, ABV. Oral PDN 5 mg	IV MEP 1 g qd (3 days). IV ACV 15 mg/Kg tid (14 days), then oral ACV 400 mg bid (4 weeks)	Improvement and subsequent stabilization
		CD4+: 536/μL					
		VL<50 c/mL					

* Microbiology: microscopic and culture for bacteria, mycobacteria and fungi; ** Viral genomes: DNA of herpes simplex virus type 1 and 2 (HSV-1, HSV-2), varicella-zoster virus (VZV), cytomegalovirus (CMV), Epstein-Barr virus, JC virus.

*VL* HIV-RNA, *VDRL* Venereal disease research laboratory test, *TPHA* Treponema pallidum hemagglutination, *neg* Negative, *CSF* Cerebrospinal fluid, *p-ANCA* Perinuclear anti-neutrophil cytoplasmic antibodies, *c-ANCA* Cytoplasmic anti-neutrophil cytoplasmic antibodies, *anti-beta 2-gp* Anti beta-2 glycoprotein, *LA* Lupus anticoagulant antibodies, *ANA* Antinuclear antibodies, *N.A.* Not available, *MRI* Magnetic resonance imaging, *Gd* Gadolinium, *FO* Fundus oculi, *RFA* Retinal fluorangiography, *VEP* Visual evoked potentials, *AEP* Auditory evoked potential, *EEG* Electroencephalogram, *cART* Combination antiretroviral therapy, *TDF* Tenofovir, *FTC* Emtricitabine, *ATV* Atazanavir, *3TC* Lamivudine, *ABV* Abacavir, *IV* Intravenous, *MEP* Methylprednisolone, *PDN* Prednisone, *IV Ig* Intravenous immunoglobulins, *GCV* Ganciclovir, *V-GCV* Valganciclovir, *ACV* Aciclovir.

**Figure 1:**
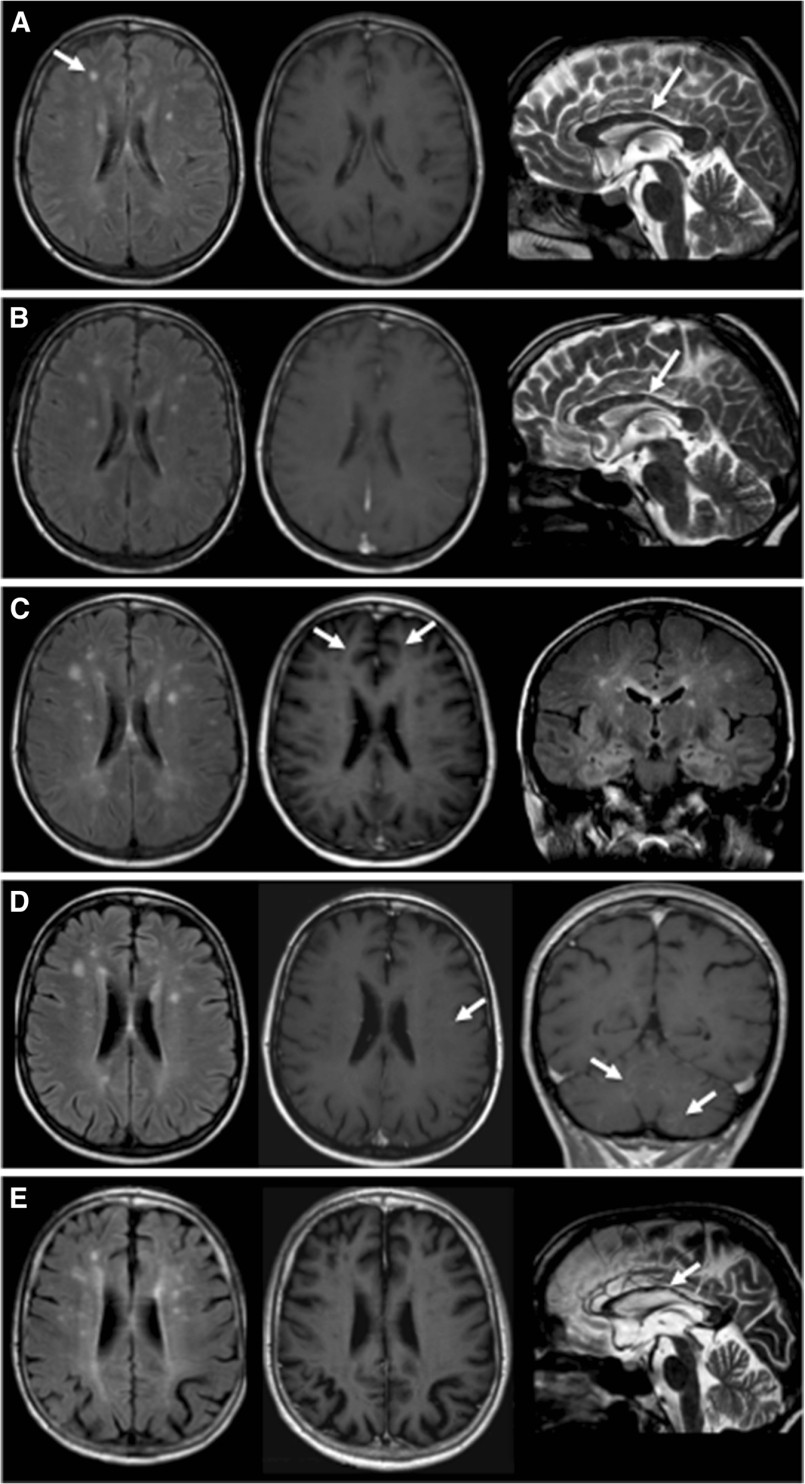
**Brain magnetic resonance imaging: axial FLAIR (first column), axial Gd -T1 (second column), sagittal T2 (third column: A, B, E), coronal FLAIR (C) or coronal Gd-T1 (D). A**. March 14th, 2008 (first relapse): T2/FLAIR hyperintense non-enhancing lesions of centra semiovalia (arrow) and corpus callosum (arrow) white matter. **B**. April 1st, 2008 (first relapse, follow-up; diagnosis of SS): increased number and intensity of the T2/FLAIR hyperintense non-enhancing lesions of brain white matter, corpus callosum (arrow), also extending to cerebellum (not shown). **C**. May 15th, 2008 (third relapse): further increase of lesion number and intensity, some of the lesions are now enhancing (arrow). **D**. November 25th, 2008 (fourth relapse): stabilization of the supratentorial lesions, but new multiple contrast-enhancing brain (arrow) and cerebellar lesions (arrows). **E**. September 6th, 2012 (long-term follow-up): no evidence of disease activity with ensuing brain atrophy, as shown by dilatation of cortical sulci and loss of brain volumes, including at the corpus callosum (arrow).

The patient remained asymptomatic for six years, with MRI showing persistence of inactive brain lesions. In January 2008 she started treatment with tenofovir, emtricitabine and unboosted atazanavir and in 4 weeks CD4+ cells increased from 202 to 260/μL and HIV-RNA dropped from 13,000 c/mL to undetectable (< 50 c/mL). In March 2008, after 6 weeks of cART, she presented with headache and paresthesias. Brain MRI showed increased T2 hyperintensity of old lesions and one new right frontal lesion, without contrast enhancement (Figure 1a). CNS-IRIS was suspected, patient was treated with oral prednisone and self-suspended cART. After three weeks, however, she was admitted to our department for worsening of previous neurological symptoms with new onset of hemiparesis and hypoacusia. Brain MRI showed further increased hyperintensity of old lesions and new non-enhancing T2 hyperintense cerebral and cerebellar lesions (Figure 1b). Fundus oculi examination showed right retinal vasculitis characterized by bilateral arteriolar wall hyperfluorescence and reduced perfusion at fluorangiography. Auditive evoked potentials confirmed the hypoacusia. SS was diagnosed based on current and retrospectively examined clinical and radiological findings along with exclusion of other central nervous system (CNS) diseases. The patient received high dose i.v. methylprednisolone with partial remission of symptoms and was discharged with maintenance oral prednisone. cART was restarted after four-weeks withdrawal.

In April 2008, three weeks after discharge, the patient was readmitted for new neurological symptoms and contrast-enhancing brain lesions at MRI (second relapse). She received high dose i.v. methylprednisolone, tapered with oral prednisone, followed by mild clinical improvement. A third relapse with new MRI contrast-enhancing lesions occurred in May 2008, while patient was still on maintenance oral prednisone (Figure 1c). She received i.v. immunoglobulin followed by 40 mg bid of i.v. methylprednisolone, with no improvement. High dose i.v. methylprednisolone was then started. I.v. gancyclovir and acyclovir were also administered for the presence of CMV pp65 antigen in plasma (5 nuclei) and suspecting a reactivation of a viral infection of the CNS, in view of the response to gancyclovir during the first episode in 2002. Patient conditions improved substantially and she was discharged with oral maintenance prednisone, acyclovir and valgancyclovir. Acyclovir was continued for 3 weeks and valganciclovir was stopped after 4 weeks for hematologic toxicity. In August 2008 cART was switched to lamivudine, tenofovir and abacavir. A fourth relapse occurred seven months later, with new MRI contrast enhancing cerebral and cerebellar lesions (Figure 1d). Patient was treated with i.v. methylprednisolone and i.v. acyclovir for 14 days, followed by clinical and MRI improvement with no further relapses.

During the following years the patient continued cART and oral prednisone maintenance at 2.5-5 mg per day, increased to 12.5-25 mg for short periods, for worsening of vertigo. She experienced no new neurological episodes, but was left with severe neurological sequelae, iatrogenic diabetes and severe osteoporosis with vertebral fractures. At last visit in January 2013 the patient had mild cognitive impairment by neuropsychological evaluation, walked with a cane and suffered from severe visual and earing loss. There was no evidence of disease activity at MRI (Figure 1e), CD4+ were 574 cells/μL and HIV-RNA <50 c/mL.

## Discussion

This is, to our knowledge, the first case of SS described in an HIV-infected person. Our patient met all the diagnostic criteria for SS, i.e., MRI findings of corpus callosum microinfarctions, leptomeningeal enhancement and grey matter involvement, evidence of branch retinal artery occlusion at retinal fluorangiography and abnormal brainstem auditory evoked potentials [1]. In agreement with what often reported [4], diagnosis was achieved only years after onset, when the classical triad of cerebral, ocular and auditory involvement was documented, along with exclusion of other etiologies.

In our case, symptoms occurred in the peculiar context of immune reconstitution, which was apparently spontaneous at the onset of SS and associated with cART in the subsequent relapses, providing the criteria for IRIS [5]. IRIS consists of a broad spectrum of inflammatory diseases that present after starting an effective cART, leading to CD4+ cells increase and, more importantly, plasma HIV-RNA reduction. [6] IRIS reflects a paradoxycal inflammatory response to opportunistic pathogens, such as *Cryptococcus spp*[7], mycobacteria [8], JC virus [9], or also to auto-antigens [10,11]. Indeed, autoimmune diseases like Graves thyroiditis, pulmunary sarcoidosis, rheumatoid arthritis, systemic lupus erythematosus and polymiositis have been reported as IRIS manifestations [10]. The recent finding of anti-endothelium cell autoantibodies in some SS patients [12] and the usually good response to immunosuppressive therapy support the autoimmune pathogenesis of SS. Thus our case suggests that SS might represent an additional manifestation of “autoimmune IRIS”.

Our patient did not show a long-lasting response to high dose i.v. steroids or i.v. immunoglobulins alone in three attempts (first and second relapses), whereas a substantial and durable improvement was achieved both times steroids were combined with antiviral drugs, i.e., acyclovir, active on herpes simplex virus type 1 and type 2 and varicella-zoster virus (VZV), and gancyclovir, also active on CMV and Epstein-Barr virus. It is known that the response to steroids in SS can not persist over time [13] and that SS can be characterized by spontaneous attenuation of the immunopathological process [3]. However, the temporal relationship between antiviral treatment and clinical improvement was strong and consistent in our case, suggesting that reactivation of an herpesvirus in the CNS might have contributed to disease pathogenesis.

We did not find evidence of herpesviruses in CSF (Table 1), but this does not exclude possible low level replication in CNS tissue [14]. We can speculate on a pathogenic model by which an herpesvirus reactivated in the context of immune reconstitution and, by replication in vascular cells [15] and consequent exposure of either viral or auto-antigens, lead to immune mediated damage of cerebral vessels. According to this model, antiviral therapy could have initially contributed to switch off the inflammatory stimulus by control of viral replication. A potential candidate herpesvirus is VZV. VZV can cause direct damage of CNS vessels of all calibres and indirect damage of CNS cells [16] and it is a well known cause of CNS vasculitis and ocular infections in HIV-infected subjects [17,18]. In addition, herpes zoster, resulting from peripheral VZV reactivation, is a frequent IRIS manifestation and cases of VZV CNS-IRIS have recently been reported [19,20].

In conclusion, our case shows that SS may occur within a dysimmune context that is possibly sustained by HIV infection, cART-associated immune reconstitution, or both. The clinical and neuroradiological remission observed when antiviral drugs were added to corticosteroids support a potential pathogenetic role of herpes virus reactivations in SS in our case.

## **Consent**

Written informed consent was obtained from the patient for publication of this Case report and any accompanying images. A copy of the written consent is available for review by the Editor-in-Chief of this journal.

## Competing interests

The authors declare that they have no competing interests.

## Authors’ contributions

FF, BC, MG, AL and PC contribuited to the care of the patient, SG to neuroradiologic examination, MT, DF and GB to laboratory analyses. FF drew up the first draft of the report, DF and PC made a substantial contribution to draft the manuscript and revised the draft. All authors read and approved the final version of the manuscript.
